# Unified rheology of vibro-fluidized dry granular media: From slow dense flows to fast gas-like regimes

**DOI:** 10.1038/srep38604

**Published:** 2016-12-07

**Authors:** Andrea Gnoli, Antonio Lasanta, Alessandro Sarracino, Andrea Puglisi

**Affiliations:** 1 Istituto dei Sistemi Complessi, CNR and Dipartimento di Fisica, Università di Roma Sapienza, P.le Aldo Moro 2 00185 Rome, Italy; 2 Departamento de Física, Universidad de Extremadura, 06071 Badajoz, Spain

## Abstract

Granular media take on great importance in industry and geophysics, posing a severe challenge to materials science. Their response properties elude known soft rheological models, even when the yield-stress discontinuity is blurred by vibro-fluidization. Here we propose a broad rheological scenario where average stress sums up a frictional contribution, generalizing conventional *μ(I*)-rheology, and a kinetic collisional term dominating at fast fluidization. Our conjecture fairly describes a wide series of experiments in a vibrofluidized vane setup, whose phenomenology includes velocity weakening, shear thinning, a discontinuous thinning transition, and gaseous shear thickening. The employed setup gives access to dynamic fluctuations, which exhibit a broad range of timescales. In the slow dense regime the frequency of cage-opening increases with stress and enhances, with respect to *μ(I*)-rheology, the decrease of viscosity. Diffusivity is exponential in the shear stress in both thinning and thickening regimes, with a huge growth near the transition.

Dry granular materials are collections of macroscopic particles, interacting through frictional contact forces^1^,^2^,^3^. The resistance of a granular aggregate to an applied shearing force is sensitive to many aspects of the experimental setup and may present analogies with macroscopic frictional laws, plasticity, soft glassy rheology and the shear thinning or thickening phenomena of suspensions^4^,^5^,^6^,^7^,^8^,^9^,^10^,^11^. Recently, consensus has been achieved on a certain class of steady slow flows which obey the so-called *μ(I*)-rheology^6^,^12^,^13^,^14^. In such a framework the shear stress *σ* is proportional to normal pressure *p* through a friction coefficient *μ(I*) = *σ/p*, which slightly depends on the shear rate itself through the adimensional “inertial number” *I*, according to the following formula:


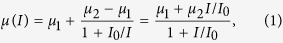


where *μ*_1_, *μ*_2_ and *I*_0_ are constants. The above formula (see red curve in Fig. 1a) expresses (at constant *p*) a monotonic growth of *σ* from a minimum yield stress *σ*_1_ = *μ*_1_*p* to a saturation (frictional) stress *σ*_2_ = *μ*_2_*p*. The inertial number 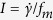 is the ratio between the shear rate 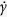 and the microscopic frequency 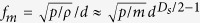 (*d* the diameter of a grain, *ρ* its material density, *m* its mass, *D*_*s*_ the space dimension). Basically *f*_*m*_ is the inverse of the time needed by a grain to move by *d* under the acceleration given by the pressure, if starting at rest. The validity of the *μ(I*) scenario has been probed in different setups and is typically associated with a dilatancy effect in the form of a *I*-dependent packing fraction *ϕ(I*)^12^. For this reason the scenario is better appreciated in experiments where the volume is not constrained. Note that Eq. (1) corresponds to a monotonic thinning-like reduction of effective viscosity 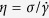 which goes from ∞ to 0 as the shear rate is increased.

A more complex picture emerges in the presence of vibro-fluidization, that is, under vertical vibration of the granular container^15^,^16^. In applications, vibro-fluidization is a renowned technique that enhances homogenization and surface of contact at the solid-gas interface for combustion chambers and chemical reactors. A parameter that characterizes the intensity of vibration is Γ = *a*_*max*_/*g*, that is the maximum vertical acceleration *a*_*max*_ (in the case of sinusoidal vibration) normalized by gravity acceleration *g* (in our experiment we have also used non-sinusoidal vibrations and therefore a more general definition of Γ, see Methods). Even at mild values of Γ (Γ < 1), an internal diffusion of kinetic energy cooperates with the applied stress and softens the discontinuities provided by enduring contacts^15^. The result is the introduction of a thermal-like energy scale (absent in non-fluidized granular media), an evident reduction of the yield stress and a faster fluidization of the material under increasing rates of deformation. Rheological studies in a split-bottom cell under vertical vibro-fluidization demonstrated the existence of a thinning transition^15^, whose exact nature is under scrutiny^17^,^18^, recently ascribed to an internal distribution of microscopic stresses and a local Herschel-Bulkley rate-stress relation^19^.

A parallel line of investigation has approached the problem of dry granular rheology by introducing the concept of partial fluidization^20^,^21^. In this context there is agreement about the hybrid nature of granular internal stress, modelled as a superposition of a frictional contribution, sustained by enduring contacts stabilized by normal pressure, and a kinetic contribution, where momentum is transferred through instantaneous collisions of the fluidized particles. The kinetic contribution is expected to be negligible in the densest and slowest regimes, while it emerges in liquid-like flows and finally becomes dominant in gas-like configurations. Notwithstanding the immediacy of the concept of partial fluidization, very different recipes and analyses have been suggested in the literature, focusing on different aspects and setups. A relevant role in this framework is played by models of non-local rheology^7^,^21^,^22^.

## Results

### A unified rheological model

**Figure 1:**
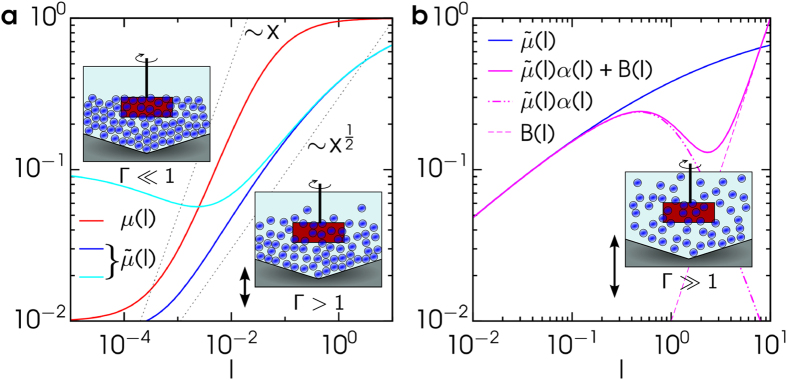
Schematic behavior of rheological functions introduced in the text: (**a**) focus on low values of *I*; (**b**) focus on larger values of *I*. In the two plots: *μ(I*) is the standard *I*-dependent friction coefficient, 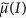 is a modified version including the effect of activated fluidization (see Eq. (3) in the text), *α(I*) is the Bernoulli pressure correction and finally *B(I*) is the Bagnold rheology function. Values of the constants are: *μ*_2_ = 1, *μ*_1_ = 0.01 in red, blue and purple curves, *μ*_1_ = 0.1 in cyan curve, *I*_0_ = 0.05, *I*_1_ = 0.001, *I*_2_ = 1, *I*_3_ = 10, *c* = 1. The three drawings represent three characteristic regimes of fluidization: the original *μ(I*) rheology describes low (or zero) fluidization, the modified 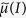 rheology includes the first effects of fluidization, the further modifications appearing in the full Eq. (2) apply to large values of Γ.

Our aim, here, is to put under scrutiny a conjecture of ours for a minimal rheological model, based upon superposition between frictional and collisional contributions to internal stresses, that can embrace the full spectrum of rotationally forced granular flows under vibro-fluidization, specifically a large range of values of *I* ∈ [10^−5^, 10] and Γ ∈ [0, 40]. In general, normal stress (pressure) *p* depends upon the degree of fluidization, i.e. upon both *I* and Γ. For this reason we take as a pressure scale *p*_00_ which is the pressure at total rest (*I* = 0 and Γ = 0): the inertial number *I* takes the same definition as above, by replacing *p* with *p*_00_. Our proposal, illustrated in Fig. 1, takes the following form for a rheological curve at constant Γ:


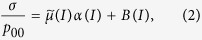


where the modified friction coefficient (blue and cyan curves in Fig. 1a) has the form


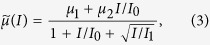


the Bernoulli pressure correction function *α(I*) (see dot-dashed purple curve in Fig. 1b) is defined as


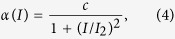


and finally the Bagnold rheology function (dashed purple curve in Fig. 1b) is simply


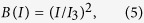


with *μ*_1_, *μ*_2_, *c, I*_0_, *I*_1_, *I*_2_ and *I*_3_ model parameters. Our proposal is not only supported by a wide agreement with experimental data, discussed below, but is substantiated through the following physical arguments.

First, in contrast with the original *μ(I*) function, a ~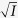 additional contribution appears at the denominator of 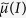: it represents “activated fluidization”, that is, the enhancement of the breakage rate of enduring contacts due to the applied stress. We note that the *I*-dependence of the friction coefficient *μ* can be ascribed to the variation of the fraction *P*_*s*_(*I*) of enduring “solid”-like contacts, namely *μ(I*) ∝ *P*_*s*_(*I*). A minimal model for *P*_*s*_(*I*) consists in neglecting memory effects (expected to be important only at very slow shear rates) and writing down a balance equation^23^ 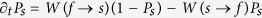, whose stationary state reads 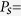 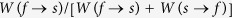
with *W(f* → *s*) and *W(s* → *f* ) the transition rates from fluid to solid state and vice-versa, respectively. Comparison with the usual *μ(I*) rheology, Eq. (1), suggests that *W(f* → *s*) and *W(s* → *f* ) are linear in *I*. On the contrary, the correction in the 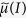, Eq. (3), implies that *W(s* → *f* ) is enhanced by an additional contribution ~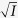. In our experiment detailed below, the analysis of fluctuations provides a transparent interpretation of such an additional term as a cage-exit frequency. The 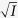-correction can also reproduce rheological behaviors of the kind shown as the cyan curve in Fig. 1a, i.e. cases of velocity-weakening (an initial reduction of 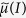 from the *μ*_1_ value) which appear in certain experiments in the absence of vibro-fluidization. Weakening cannot be explained by the usual *μ(I*) function, which is necessarily monotonic. The *α(I*) correction to pressure 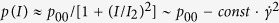 dictates the drop in pressure in the presence of finite fluid velocity, in analogy with classical Bernoulli’s principle. Finally, the Bagnold rheology function *B(I*) provides us with the inertial contribution of instantaneous collisions, dominating at large *I*, where one expects a viscous contribution *σ* ~ *γ(I)I* and the “thermal” fluctuations underlying effective viscosity are ruled by the shear rate itself, that is *γ* ~ *I*. The Bagnold relation is usually indicated as a case of shear thickening, even if there is no universal consensus on whether the word “thickening” should be reserved for dense suspensions, or it also applies to inertial effects arising in diluted fluids.

Gathering all the pieces together, a general rheological curve is obtained, an instance of which is shown as solid purple curve in Fig. 1b. At the transition between the solid-dominated and the kinetic-dominated regions it is possible to observe a non-monotonic van der Waals-like behavior of *σ* which, in stress-controlled experiments, appears as a discontinuous thinning^15^,^18^. It is straightforward to verify that a continuous change of parameters appearing in Eq. (2) transforms the non-monotonic crossover in a monotonic one, as seen in the experiments. We underline that the non-monotonic crossover between 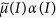 and *B(I*) is clearly distinct from the velocity-weakening effect discussed above, which belongs to the behavior of 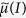 alone.

The unified rheological formula, Eq. (2), contains a series of parameters which depend, among other physical aspects of the setup, upon the intensity of vibro-fluidization Γ. We remark that in the frictional contribution 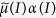 the dependence on Γ is expected to have a behavior opposite to that in the kinetic contribution *B(I*). Indeed, vibro-fluidization reduces the steady fraction of enduring contacts, while increasing the thermal agitation of flying/colliding particles. Such contrasting dependencies neatly reflect our experimental observations, as described below.

### Comparison with experiments

**Figure 2:**
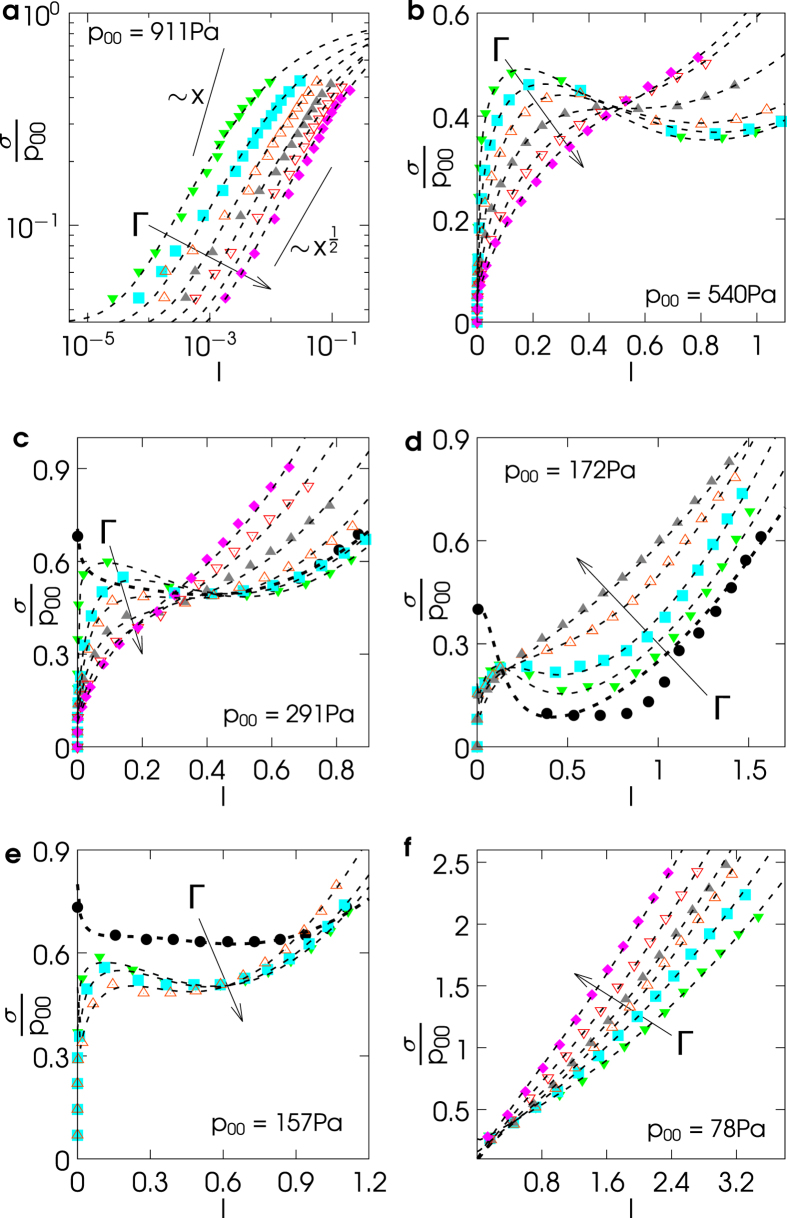
Experimental stress-strain flow curves. Each series of data with the same colour belongs to a value of the shaking amplitude Γ. In frame (**a**) the results are obtained with *N* = 2600 spheres of steel, with values of Γ = 3.4, 6.7, 11.6, 18.3, 27.4, 38.4 (from green to purple). In frame (**b**) *N* = 1300 spheres of steel, with values of Γ = 2.4, 5, 8.9, 14.6, 22.5, 31.9 (from green to purple). In frame (**c**) *N* = 2600 spheres of glass, with values of Γ = 0 (black) and Γ = 1.1, 8.7, 14.3, 22.1, 32, 43 (from green to purple). In frame (**d**) *N* = 1300 spheres of glass, with values Γ = 0 (black) and Γ = 9, 14.7, 22.5, 32.4 (from green to gray). In frame (**e**) *N* = 2600 spheres of delrin, with values Γ = 0 (black) and Γ = 0.8, 1.3, 3.7 (from green to gray). Finally, frame (**f**) displays the results of *N* = 600 spheres of steel, with values of Γ = 6.9, 8.6, 10.7, 13.2, 19.2, 26.2 (from green to purple). Dashed lines are best fits with Eq. (2). The values of the fits’ parameters are given in Supplementary Table S2.

The theoretical picture of Eq. (2) fairly describes the broad phenomenology observed in the experiments we carried out. These are inspired by vane-test tools for the *in*-*situ* rheology of soils^24^,^25^, while the granular medium undergoes mechanical vibro-fluidization in the vertical direction. Experiments are detailed in the Methods section. The observed rheological curves *σ* vs *I* explore ranges of *I* which depend upon *p*_00_. The six frames in Fig. 2 show several representative cases together with their best fits through Eq. (2).

Frame (*a*) illustrates a case at high *p*_00_ which provides us with a high resolution at low 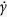, i.e. zooming in the first part of Eq. (2), where the kinetic contribution is negligible and *α(I*) ~ *c*. The ~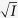 behavior is evident, as well as a small but non-negligible yield stress *μ*_1_ > 0. At intermediate values of *p*_00_ (frames (*b*), (*c*), (*d*) and (*e*)) the flow curve *σ* vs *I* exhibits the crossover from the solid-dominated regime to the collisional-dominated regime, which at low Γ is non-monotonic. Increasing Γ the parameters change continuously, leading to a point where the curve becomes monotonic. The pressure at rest in cases (*c*), (*d*) and (*e*) is low enough to allow a series of data at Γ = 0 (see black circles) where a large yield stress can be measured. Finally, frame (*f*) reports a low pressure situation, where the collisional part of Eq. (2) dominates, leading to thickening-like behavior, that is an increasing differential effective viscosity ∂*σ*/∂*I*. The six frames confirm what we argued in the above theoretical discussion: when the stress is dominated by the solid contribution, an increase of Γ leads to a reduction of stress, while the opposite occurs when the kinetic contribution dominates. At a given *p*_00_, the value of *I* corresponding to the crossover between the two regimes does not depend upon Γ: indeed the non-monotonic curves (cases (*b*), (*c*), (*d*) and (*e*) at low Γ) cross, roughly, at a single point.

In Supplementary Section S1 we discuss the systematic behavior of the fit parameters (which are reported in Supplementary Table S2). The physical consistency of the model is corroborated by a general smoothness of parameters’ behavior when Γ is increased. It is more difficult to find a clear connection between the values of the parameters and *p*_00_ (controlled by material density and by *N*), an aspect which certainly deserves further investigation.

### Microrheological properties

Further support for our picture comes from the study of fluctuations, made feasible by our vane-test experiment where the rotating blade behaves also as a micro-rheological probe^26^,^27^,^28^. In particular we have measured diffusivity 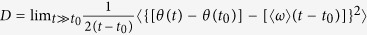 (where *θ(t*) is the angular position of the blade), the frequency of relaxation of the angular velocity *ω(t*) defined as *f*_*ω*_ = 〈(*ω* − 〈*ω*〉)^2^〉/*D*, and the frequency of typical cage exit *f*_*cage*_ (which is well defined only in the slow dense cases at high *p*_00_^26^). The precise definition of those quantities is given in the Methods section.

**Figure 3:**
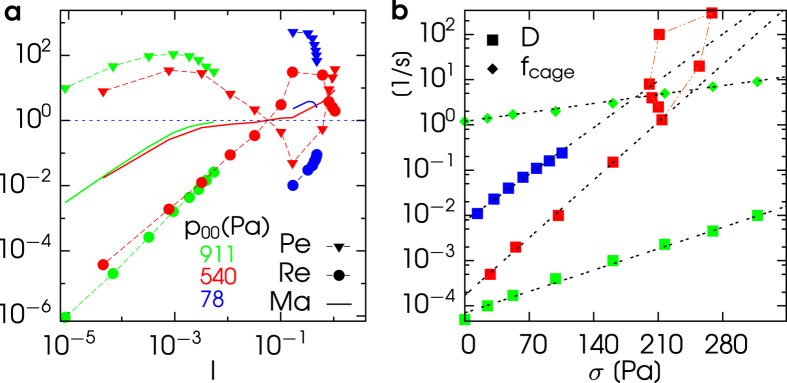
Frame (**a**) Peclet, Reynolds and Mach numbers, as functions of the inertial number *I*, in experiments at *p*_00_ = 911 *Pa* (green symbols and lines, 2600 spheres of steel shaken at Γ = 3.4), at *p*_00_ = 540 *Pa* (red symbols and lines, 1300 spheres of steel shaken at Γ = 2.4), and at *p*_00_ = 78 *Pa* (blue symbols and lines, 600 spheres of steel shaken at Γ = 10.7). Frame (**b**) diffusivity *D*, for all three experiments as in frame (**a**), and cage-exit frequency *f*_*cage*_ (only for experiment at *p*_00_ = 911 *Pa*), as function of the average measured stress *σ*. In frame (**b**) the dashed lines represent exponential fits.

In order to identify the relevant physical quantities responsible for the different regimes observed in our system, we define the Péclet number Pe = 〈*ω*〉/*D*, the Reynolds number Re = 〈*ω*〉/*f*_*ω*_ and the Mach number 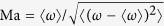, which are shown in Fig. 3, frame (a), for a set of experiments. For not too high values of the inertial number *I*, we find that, in both cases at high and intermediate *p*_00_, 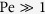 (green and red triangles) and 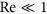 (green and red circles): this behavior can be therefore interpreted as a regime where damping dominates over diffusion.

In the opposite limit, at high values of *I*, for the case *p*_00_ = 540, we find both Pe and Re > 1, with Pe > Re. This regime corresponds to the case where inertia dominates, leading to an increase of friction with a consequent thickening behavior, in agreement with the Bagnold contribution appearing in our phenomenological model, and with the general picture presented in ref. 29. Interestingly, in the intermediate regime, an inversion occurs - with Pe becoming smaller than 1 and Re larger than 1 - at a value of *I* corresponding to Ma crossing 1, comparable to that where the unstable branch of *σ(I*) begins.

For the low pressure data, *p*_00_ = 78, we again find 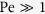 (blue triangles) and 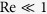 (blue circles), i.e. a regime dominated by damping. The measure of the Mach number allows us to distinguish between the cases at high and low pressure: indeed we find the crucial difference that Ma < 1 in the high *p*_00_ case (green line) whereas Ma > 1 in the low *p*_00_ case (blue line). This result reflects the observation that in the latter case the vane can drag the surrounding granular medium.

Green diamonds in Fig. 3b indicate *f*_*cage*_ ~ exp(*σ*) which at low values of stress is well approximated by *f*_*cage*_ ~ 1 + *σ*. This observation, together with the behavior *σ* ~ 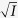 seen in Fig. 2a, is compatible - at low rates *I* - with our interpretation of the denominator of 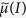: the main responsible factor for the loosening of solid-like contacts is the activated escape from trapping cages^30^. A further observation concerns the dependence of *D* on *σ*, again displayed in Fig. 3b: in all regimes, excluding the dense-dilute crossover region, we observe a striking exponential behavior *D* ~ exp(*σ*). This law seems universal and denotes a wide variability of *D* when *σ* is varied keeping Γ constant. For instance in cases near the transition a variation of more than three decades appears. Those findings reveal an extreme sensitivity of micro-dynamics to external disturbances which is critical in designing industrial processes or predicting geophysical hazards.

## Discussion

We have introduced a novel rheological model, Eq. (2), which merges a corrected *μ(I*)-like frictional contribution dominating at small *I* and a Bagnold-like term which gives high velocity thickening effects (Eq. (5)). The frictional contribution is modified to take into account the fact that the cage-opening rate is *I*-dependent (Eq. (3)), and that (particularly in vibrofluidized experiments) the pressure due to enduring contacts is reduced with increasing *I* (Eq. (4)). The unifield model is able to describe the softening of yield stress with vibro-fluidization, velocity weakening, shear thinning, the often observed discontinuos thinning transition, and shear thickening at large *I*. We have employed a vibrofluidized vane setup to reproduce most of the mentioned phenomena and compare the flow curves with Eq. (2), confirming its wide applicability. A microrheological study of experimental fluctuations has offered further insight, giving solid arguments to the cage-opening interpretation of the ~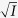 correction to the standard *μ(I*) formula. We have also observed a striking sensitivity of diffusivity to the shear stress.

## Methods

### Details of the experiment

The granular medium was made of a number *N* ∈ [300, 2600] of spheres of diameter *d* = 4 mm made of non-magnetic steel (mass of each sphere: 0.267 g), glass (mass 0.0854 g), or delrin^®^ (mass 0.0462 g). They were housed in a plexiglas^®^ cylinder with a conical-shaped floor (diameter 90 mm, minimum height 28.5 mm, maximum height 47.5 mm) in which a plexiglas vane (height 15 mm, width 6 mm, length 35 mm) was suspended in order to be in contact with the granular medium and not with the container^31^,^32^,^33^. The container was vertically vibrated by an electrodynamic shaker (LDS V450) fed with an acceleration signal *a(t*). In most of the experiments *a(t*) is a white noise with a band-pass filter between 200 Hz and 400 Hz, while in the lowest *p*_00_ case (*p*_00_ = 78 Pa) we used a sinusoidal signal at frequency 53 Hz. This choice is motivated by two empirical observations: 1) a lower number of particles (as in the case of low *p*_00_) requires a larger energy input to be homogeneously fluidized and to reach the blade, and this can be obtained by supplying energy through a sinusoidal signal at low frequency; 2) in dense cases a sinusoidal signal induces spurious resonances, while in diluted cases such resonances are never observed. We have checked that performing the same experiments with noise signal for *a(t*) (pushing the shaker to its working limits) gives flow curves with the same shape. An accelerometer placed on the container side measured *a(t*), allowing us to define 
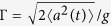
. The vane, mounted through its rotation axis to a rotary encoder, was also connected to a dc motor (typical working voltage 12 V) as the source of the driving torque. The motor was directly fed by a dc voltage supply in the range 0 to 7 V. No limit was set for the maximum current absorbed by the motor that, averaged on the duration of the experiment, was never higher than 450 mA. A data acquisition system collected data for the angular position/velocity of the vane, the effective motor voltage, the current circulating in the motor and the root-mean-square vertical acceleration of the container. A procedure of calibration allowed us to translate average values of current into average values of applied torque. The same procedure helped determining the moment of inertia of the rotating block, 3.2 × 10^2^ g mm^2^ (the blade with its axis and the gears linking it to the motor). The typical experiment, at a given Γ and applied motor voltage, was 3600 s long, with the granular always “reset” at the beginning of each run for 30 s at high shaking intensity (Γ = 42) and motor off. This procedure - together with periodic replacement of used spheres - guaranteed reproducible results at a distance of several weeks. Packing fractions was non-homogeneous (it was larger in regions far from the borders of the container): its value at rest was estimated to be in the range 55–70%, while it decreased when vibration was switched on. In the analysis we have identified the shear rate 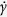 with the average of the angular velocity *ω(t*) of the rotating blade, i.e. 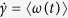, while the shear stress *σ* is proportional to the average of the applied torque *T(t*) through the shear stress constant *κ*, i.e. *σ* = *κ*^−1^〈*T(t*)〉 with *κ* = 2*πR*^2^*H* (with *R* and *H* the blade half-length and height, respectively)^24^.

### Details of data analysis

Velocity power density spectra (VPDS) are defined as 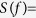 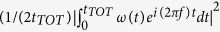 with *t*_*TOT*_ the time-length of an experiment (=3600 seconds). Some examples of *S(f* ) curves are shown in the Supplementary Fig. S4. In ref. 26 VPDS in a similar vibro-fluidized experimental setup, without applied torque (*σ* = 0), has been investigated. In the dilute or gas-like limit, e.g. low number of spheres at high shaking, the VPDS takes a simple Lorentzian shape *S(f* ) = *D*/[1 + (2*πf/f*_*visc*_)^2^], with *D* the asymptotic (long time) diffusivity and *f*_*visc*_ the effective viscosity due to granular gas-vane collisions. When the number of particles (density) is increased and/or the intensity of shaking (Γ) is reduced, the system approaches a slow liquid regime and the VPDS develops a wide bump (or smooth peak) with a maximum near *f* ~ 20 Hz, which is associated to oscillations of the velocity autocorrelation induced by liquid cages. At much smaller frequencies the VPDS reaches a plateau whose height, lim_*f*→0_*S*(*f*), corresponds to diffusivity *D*: indeed the blade is not trapped in a cage forever, eventually it manages to explore a much larger phase space and reaches normal diffusion. From the low frequencies plateau of VPDS we have extracted values of *D* for Fig. 3. We have defined the cage-exit frequency *f*_*cage*_ as the x-position, in the VPDS plot, of the minimum separating the cage bump from the low-frequency diffusive plateau (see filled circles in Supplementary Fig. S4).

### Data availability

The data that support the plots within this paper and all results reported in this study are available from the corresponding author upon request.

## Additional Information

**How to cite this article**: Gnoli, A. *et al*. Unified rheology of vibro-fluidized dry granular media: From slow dense flows to fast gas-like regimes. *Sci. Rep.* **6**, 38604; doi: 10.1038/srep38604 (2016).

**Publisher's note:** Springer Nature remains neutral with regard to jurisdictional claims in published maps and institutional affiliations.

## Supplementary Material

Supplementary Information
