# Supplementary material for: Unified rheology of vibro-fluidized dry granular media: From slow dense flows to fast gas-like regimes
Source: Sci Rep. 2016 Dec 7;6:38604. doi: 10.1038/srep38604 (PMC5141475; doi:10.1038/srep38604)
Supplement: Supplementary Information [file srep38604-s1.pdf]

Unified rheology in vibro-fluidized dry granular media: From slow dense flows to fast gas-like regimes. SUPPLEMENTARY INFORMATION

Andrea Gnoli[1], Antonio Lasanta[1,2], Alessandro Sarracino[1], and Andrea Puglisi[1]
[1]*Istituto dei Sistemi Complessi - CNR and Dipartimento di Fisica, Università di Roma Sapienza, P.le Aldo Moro 2, 00185, Rome, Italy*
[2]*Departamento de Física and Instituto de Computación Científica Avanzada (ICCAEx), Universidad de Extremadura, 06071 Badajoz, Spain*

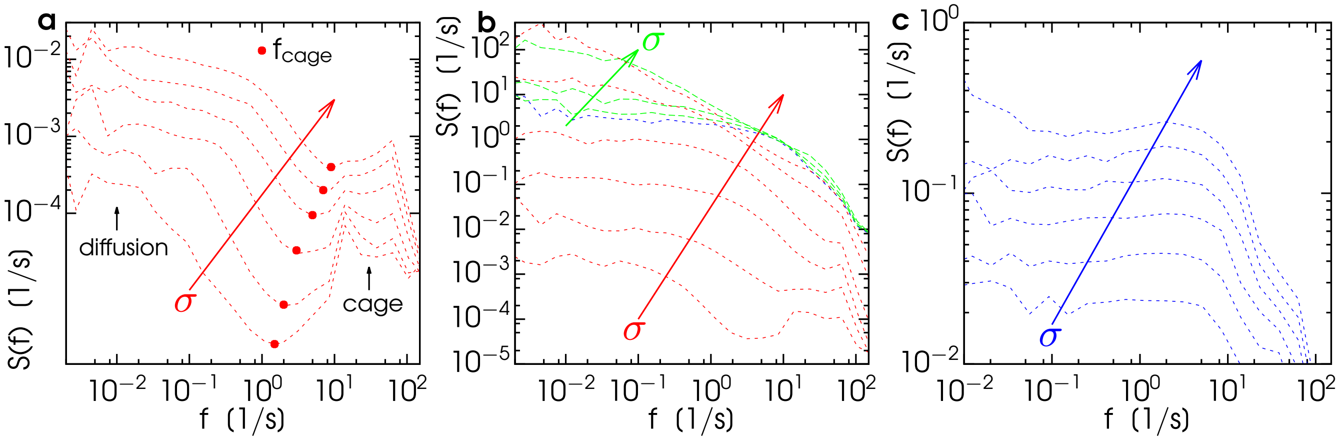

FIG. S4. Power density spectra for three different values of p_{00} at mild shaking (same experiments as in Fig. 3 of main text): a) a case with 2600 spheres of steel ($p_{00} = 911$ Pa) at $\Gamma = 3.4$, b) a case with 1300 spheres of steel ($p_{00} = 540$ Pa) at $\Gamma = 2.4$, c) a case with 600 spheres of steel ($p_{00} = 78$ Pa) at $\Gamma = 10.7$.

S1. PARAMETERS FOR THE FITS OF RHEOLOGY CURVES.

In Table S2 we report the values of the parameters for the fits of experimental data (Fig. 2 of main text) through Eq. (2) of main text (errors are on the last digit). Let us comment on the general trends of the fit parameters in our different experiments.

We first note that the relative value of the yield stress, μ_1/μ_2, decreases upon increasing Γ in all cases but the one at $p_{00} = 157$ (where it is almost constant). This means that one effect of the shaking is to enlarge the difference between the minimum yield stress and the saturation (frictional) value. At fixed Γ, the ratio μ_1/μ_2 shows a growing behavior upon decreasing p_{00} (except for the very dilute case at $p_{00} = 78$), and reaches values around ~ 0.7 for $p_{00} = 157$. Therefore the jump in yield stress is reduced at low pressures.

The parameters I_0 and I_1 increase with Γ, with a power law $I_0, I_1 \sim \Gamma^2$, for all pressures apart from the low-pressure cases, $p_{00} = 172$ and $p_{00} = 78$, where the behavior is less clear. The value of $c\mu_2$ is almost constant in all cases, and shows a slight increase, in particular for $p_{00} = 78$. I_2 does not exhibit a strong dependence on Γ, except for the cases $p_{00} = 172$ and $p_{00} = 78$, while it shows a non-monotonic behavior with p_{00}. Finally, I_3 slightly decreases upone increasing Γ and is non-monotonic with p_{00}.

S3. ANALYSIS OF VELOCITY POWER DENSITY SPECTRA.

In Figure S4 we show some of the power density spectra of the angular velocity time-series $\omega(t)$ measured by the blade in 3600 seconds-length experiments. The velocity power density spectrum is defined as

$$S(f) = \frac{1}{2t_{TOT}} \left| \int_0^{t_{TOT}} \omega(t) e^{i(2\pi f)t} dt \right|^2 . \tag{1}$$

In frame (a), reporting results for the high density/pressure and low velocity experiments, we have marked with "cage" the bump - in the region $10 - 100$ Hz, associated to fast elastic oscillations related to trapped dynamics. In the same frame, the frequency of cage-exit (f_{cage} in the main text) is the ascissa of the filled dot. At low frequencies the height of the characteristic plateau, present in all experiments, defines the diffusivity D. In all frames the arrow represents the order of growing σ (shear stress or average applied torque). In the central frame, the green curves represent the values of σ which decrease when $\dot{\gamma}$ increases (see green triangles in Fig. 2b of main text). The blue curve corresponds to the first point where σ starts to grow again at large $\dot{\gamma}$.

Γ	μ_1/μ_2	I_0	I_1	$c\mu_2$	I_2	I_3
p_{00} = 911 Pa (N = 2600 spheres of steel)						
3.40	0.040	0.001	$1.2{\cdot}10^{-4}$	0.95	∞	∞
6.70	0.037	0.003	$3.6{\cdot}10^{-4}$	0.95	∞	∞
11.6	0.033	0.007	$7.0{\cdot}10^{-4}$	1.03	∞	∞
18.3	0.030	0.015	0.0015	1.08	11.11	∞
27.4	0.022	0.035	0.0030	1.36	10.00	∞
38.4	0.020	0.050	0.0032	1.44	8.33	∞
p_{00} = 540 Pa (N = 1300 spheres of steel)						
2.4	0.07	0.0020	$4.0{\cdot}10^{-4}$	0.65	0.65	2.27
5.0	0.04	0.0055	$5.0{\cdot}10^{-4}$	0.75	0.69	2.35
8.9	0.04	0.039	0.0080	0.98	0.65	2.21
14.6	0.04	0.10	0.0153	1.25	0.67	1.89
22.5	0.05	0.32	0.23	1.21	0.71	1.61
31.9	0.04	0.50	0.50	1.25	0.80	1.59
p_{00} = 291 Pa (N = 2600 spheres of glass)						
0	0.47	0.85	0.38	1.50	0.61	1.27
1.1	0.29	$1.1{\cdot}10^{-4}$	$2.1{\cdot}10^{-5}$	0.66	0.53	1.29
8.7	0.15	0.026	0.795	0.69	0.53	1.25
14.3	0.12	0.029	0.80	0.62	0.53	1.11
22.1	0.17	0.045	0.80	0.55	0.67	1.02
32.0	0.15	0.070	0.80	0.55	0.77	0.91
43.0	0.12	0.174	28.8	0.67	0.67	0.81
p_{00} = 172 Pa (N = 1300 spheres of glass)						
0	0.9	$3{\cdot}10^{-6}$	$3{\cdot}10^{-7}$	0.43	0.14	2.04
9	0.25	0.041	0.011	0.63	0.26	1.89
14.7	0.22	0.06	0.007	0.78	0.37	1.75
22.5	0.25	0.015	0.0053	0.35	1.14	1.72
32.4	0.24	0.013	0.00016	0.74	10	2.13
p_{00} = 157 Pa (N = 2600 spheres of delrin)						
0	0.53	0.61	0.55	1.5	0.88	1.75
0.8	0.6	0.0002	$5.5{\cdot}10^{-5}$	0.62	0.69	1.49
1.3	0.73	0.001	0.00016	0.69	0.65	1.45
3.7	0.66	0.0067	0.0013	0.76	0.59	1.33
p_{00} = 78 Pa (N = 600 spheres of steel)						
6.9	0.083	3.90	1.44	3.21	16.13	3.13
8.6	0.062	2.35	1.36	2.59	9.09	2.78
10.7	0.034	2.69	1.71	3.00	10.31	2.56
13.2	0.026	2.55	1.75	3.15	7.69	2.44
19.2	0.022	3.50	2.66	4.40	7.14	2.35
26.2	0.017	4.80	4.18	6.43	7.69	2.27

TABLE S2. Table of parameters for the fits of Figure 2.
